# Effects of AMF Compound Inoculants on Growth, Ion Homeostasis, and Salt Tolerance-Related Gene Expression in *Oryza sativa* L. Under Salt Treatments

**DOI:** 10.1186/s12284-023-00635-2

**Published:** 2023-04-10

**Authors:** Bo Zhang, Feng Shi, Xu Zheng, Hongyang Pan, Yuqiang Wen, Fuqiang Song

**Affiliations:** 1 Engineering Research Center of Agricultural Microbiology Technology, Ministry of Education & Heilongjiang Provincial Key Laboratory of Ecological Restoration and Resource Utilization for Cold Region & Key Laboratory of Microbiology, College of Heilongjiang Province & School of Life Sciences, Heilongjiang University, Harbin, 150080 China; 2 Jiaxiang Industrial Technology Research Institute of Heilongjiang University, Jiaxiang, 272400 Shandong China

**Keywords:** Rice, AMF compound inoculants, Salt stress, Ion homeostasis, Gene expression

## Abstract

Increased soil salinization is among the main factors that limits safe rice production. Arbuscular mycorrhizal fungi (AMF) have been shown to alleviate the toxic effects of salt stress in plants. However, more studies on AMF combined with other functional microorganisms are needed to further improve salt tolerance in rice. Therefore, the compound inoculum *Funneliformis mosseae* (Fm) together with two functional microorganisms, *Piriformospora indica* (Pi) and *Agrobacterium rhizogenes* (Ar) was evaluated for their effect on the rice growth, photosynthetic gas exchange parameters, ion homeostasis, and the expression of salt tolerance-related genes under 0, 80, 120 and 160 mM salt stress conditions. The results showed that: (1) the rice seedling biomass of the AMF compound inoculant treatment group was significantly higher than that of the non-inoculation treatment group (*P* < 0.05); (2) under NaCl stress, inoculation with AMF compound inoculants can activate the rice antioxidant enzyme system and improve osmoregulation ability; (3) AMF compound inoculants can increase the concentration of K^+^ in the plant and inhibit the transfer of Na^+^ to rice leaves, maintaining a high K^+^/Na^+^; and (4) AMF compound inoculants could induce and regulate the overexpression of genes related to salt tolerance, photosynthesis and ion homeostasis in rice, and improve the tolerance of rice under salt stress. Our study showed that AMF compound inoculants could improve the adaptability of rice under NaCl stress and promote plant growth by regulating the photosynthetic gas exchange parameter, reactive oxygen species (ROS) scavenging ability, and ion homeostasis of plants. These results suggest that AMF compound inoculants may play an important role in improving rice productivity in salinized soil.

**Graphical Abstract:**

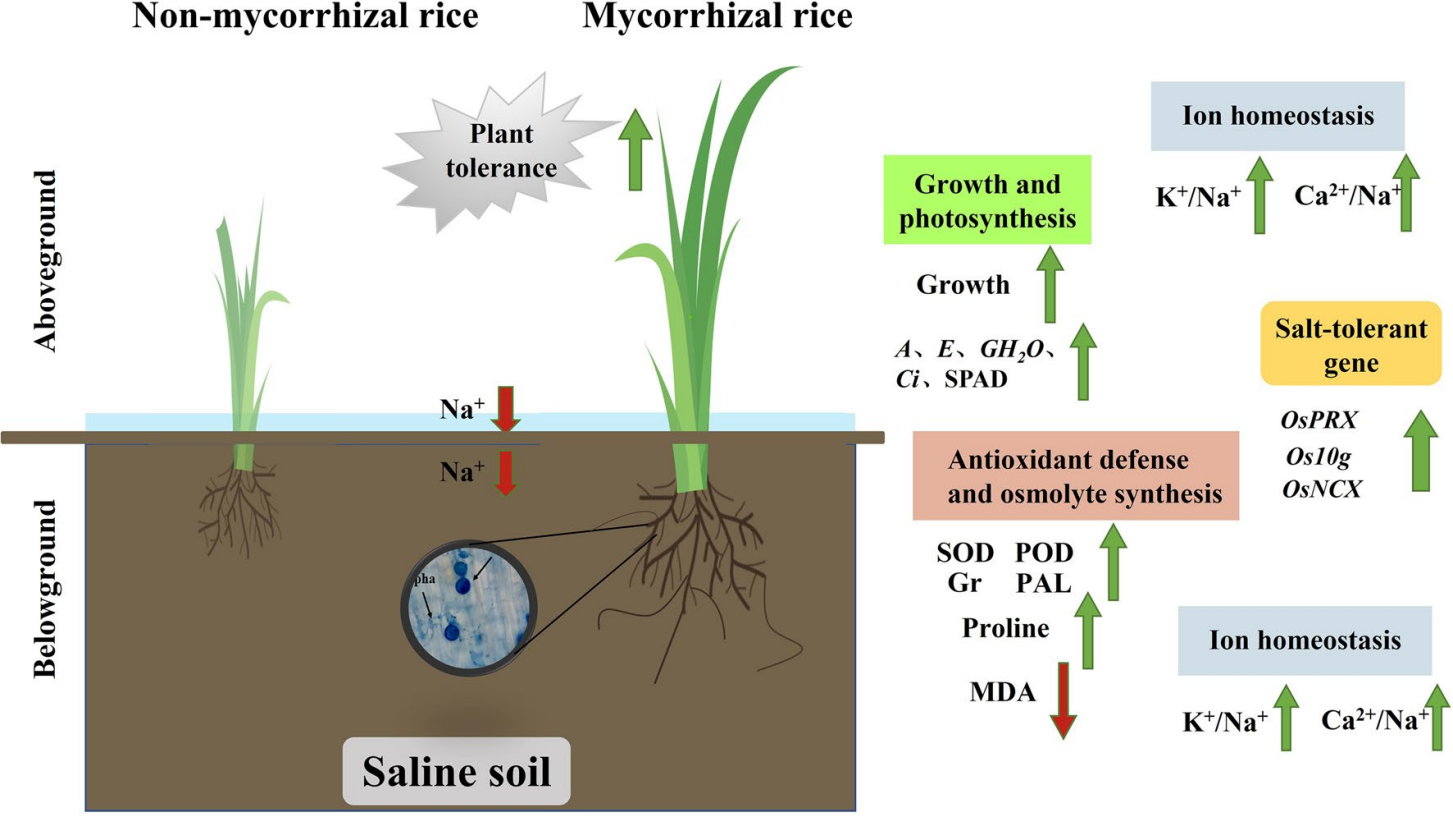

**Supplementary Information:**

The online version contains supplementary material available at 10.1186/s12284-023-00635-2.

## Introduction

The world’s population has grown rapidly over the past two decades, leading to a sharp increase in the global demand for crop yields (Savary et al. 2020). How to improve the yield and quality of crops has become an urgent global agricultural problem. Rice (*Oryza sativa* L.) is among the leading food crops worldwide, and its planting area is extensive. Rice yield is of great significance to the development of the world population (Zeng et al. 2001). Salt stress is an important abiotic stress factor that threatens plant growth and development; in addition, salt stress is a severe problem that restricts agricultural and forestry production and ecological environment construction (Hazell and Wood 2008). It is estimated that approximately 20% of irrigated soils worldwide are affected by different degrees of salinization, and the soils are continually deteriorating due to inappropriate crop irrigation measures, excessive fertilization, unreasonable farming methods, rising sea levels leading to salt intrusion into coastal areas, etc. (Kamran et al. 2019). Rice is generally sensitive to salt stress, which can slow rice growth and reduced yields (Ahmed et al. 2021). Thus, improving the adaptability of rice to increasingly salinized soil is clearly significant for effectively increasing rice yield to meet the growing food demand.

Salt stress mainly causes damage to plants through osmotic stress and ion toxicity (Quan et al. 2007). When the soil salinity increases, the water potential of the soil solution becomes lower than the water potential of the plant root cells, resulting in osmotic stress. As a result, the roots cannot absorb water and results to plants experiencing a physiological shortage of water (Razzaq et al. 2020). Osmotic stress causes stomatal closure, which inhibits plant uptake of CO_2_ and leads to a decrease in photosynthesis (Qin and Huang 2020). Plants must perform osmoregulation and produce many osmotic adjustment substances, such as proline (Pro) in the cells to maintain the normal expansion, growth, and water absorption of cells (Liu et al. 2021a). Water deficiency in plants alters the intercellular osmotic pressure and reduces the efficiency of nutrient transport, leading to nutrient deficiency (Chen et al. 2020). On the other hand, ionic stress is mainly caused by the massive accumulation of Na^+^ in the soil in plant cells (Yang and Guo 2018). Excessive Na^+^ inhibits the activity of various enzymes in plants and interfere with the uptake of K^+^, Ca^2+^, Zn^2+^, and other elements by plants (Wu et al. 2018; Iqbal et al. 2018). Salt stress results to the production of large amounts of reactive oxygen species (ROS) in plants, which can impact plant growth and development and even lead to plant death (Parihar et al. 2020). Plants usually eliminate ROS by producing many antioxidant enzymes such as superoxide dismutase (SOD) and peroxidase (POD), resisting salt stress and alleviating the damage caused by salt stress (Shehab et al. 2022). Salt stress not only affects the growth and yield of rice but also affects the quality of rice. At present, much attention is being placed on the development of new biotechnology methods to improve the stress resistance of rice and alleviate the damage caused by salt stress.

Arbuscular mycorrhizal fungi (AMF) is an endophytic fungus that is widely distributed in nature and can form symbioses with 80% of terrestrial plants; in addition, AMF are the class of fungus most closely related to agricultural production in the soil microflora (Hashem et al. 2018). AMF improve the nutritional status of the host plant by establishing a symbiotic relationship with the host and helping the host plant absorb water and nutrients from the soil (Abdelhameed et al. 2018). *Funneliformis mosseae* (Fm) can greatly improve the salt tolerance of host plants by increasing host plant biomass, enhancing photosynthetic intensity, antioxidant enzyme activity, and osmoregulatory capacity (Qin and Huang 2020; Shahvali et al. 2020; Santander et al. 2021; Wang et al. 2020a, b). AMF applications show great potential for improving rice yield and enhancing rice salt tolerance. *Piriformospora indica* (Pi) can improve the tolerance of crops to adverse stress and induce plant systemic resistance (Singh et al. 2000). When used in combination with AMF, the specific functions of both fungi can be further exploited. Moreover, there is no competition between the combined microorganisms. (Moreira et al. 2015). *Agrobacterium rhizogenes* is a kind of soil bacteria that can infect a wide variety of organisms; it can infect plants and induce plants to promote plant rooting and hairy root differentiation. In addition, *Agrobacterium rhizogenes* is also a kind of mycorrhizal helper bacteria, that contributes to the colonization of AMF in rice roots (Frey et al. 2007). We used two fungi and one bacterium to create AMF compound inoculants. Through rice field planting experiments, we found that AMF compound inoculants promoted the colonization of two endophytic fungi in rice roots, and exhibited a stronger effect on the growth and development of rice (Zhang et al. 2022). In this regard, we hypothesized that AMF compound inoculants could increase the expression of relevant genes in rice seedlings, resulting in increased biomass, antioxidant enzyme activity, and osmoregulatory substance content of rice seedlings. The inoculants can regulate the cation concentration in rice seedlings and improve salt tolerance in rice by the above means.

In this study, we investigated the effects of AMF compound inoculants on the biomass, photosynthetic gas exchange parameters, antioxidant enzyme activities, as well as the dynamic balance of Na^+^, K^+^, and Ca^2+^ ions in shoots and roots of rice seedlings grown under 0, 80, 120 and 160 mM salt stress conditions. The relative differential expression of some relevant salt tolerance-related and photosynthesis-related genes was also quantified through qRT-PCR. The aim of the study was to explore some of the AMF-mediated plant responses that improve the salt tolerance of rice seedlings which supports the practical application of AMF compound inoculants for rice cultivation in salinized soils.

## Results

### Effect of Salt Stress on AMF Inoculation and Spores in the Rhizosphere Soil of Rice

**Fig. 1:**
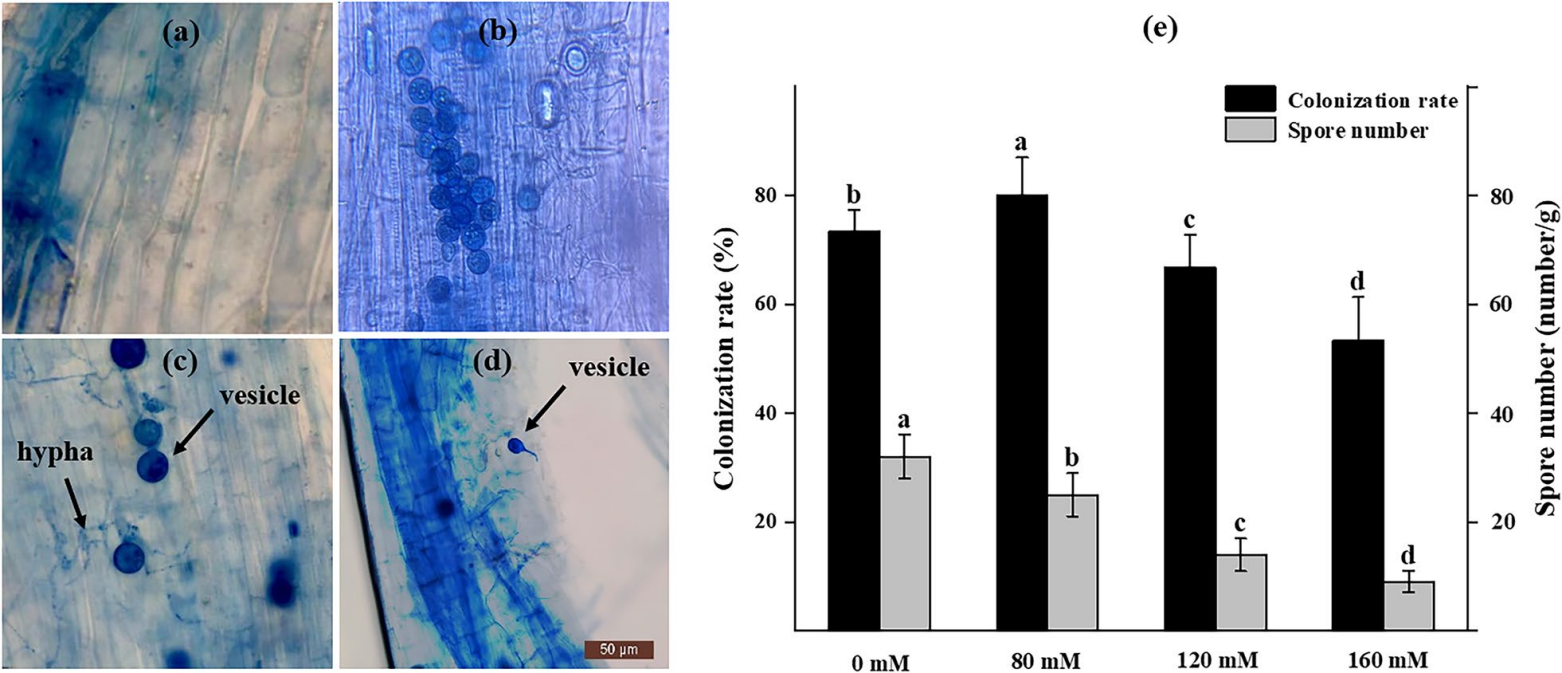
The rice root colonization of AMF under the microscope (400x) and their effective colonization in terms of colonization rate and spore number. Note: **a** nm treatment group; **b** AMF spores in rhizosphere soil; **c** Fm + Pi + Ar treatment group; **d** Fm + Pi + Ar treatment group; (e) Rice mycorrhizal colonization rate and the number of spores in the rhizosphere soil. The results are presented as the mean ± standard deviation of three replicates. Different letters indicate differences between different treatments (*P* < 0.05)

AMF compound inoculants established symbiotic relationship with rice. Figure 1 bcd shows that the roots of rice possess clear vesicles, hyphae, and Pi spore structures. However, fungal infection was not detected in the nm treatment group, which indicated the absence of indigenous AMF and Pi in the soil matrix (Fig. 1 a). By mycorrhizal infection rate determination, we found that the mycorrhizal infection rate of the am 80 treatment was up to 80% (Fig. 1 e); and was significantly different (*P* < 0.05) from the am 0 treatment. The growth rate was 9.1%, however, as the concentration of salt stress continued to increase, the root infection rate decreased significantly (*P* < 0.05). Salt stress treatment significantly reduced the number of spores in rice rhizosphere soil (*P* < 0.05). Compared with the am treated rice roots at 0 mM, the spore number in the roots of rice grown under 80 mM, 120 mM, and 160 mM salt stress decreased by 21.9%, 56.3% and 71.9%, respectively.

### Effects of Salt Stress on Photosynthetic Gas Exchange Parameters and SPAD of Rice

**Fig. 2:**
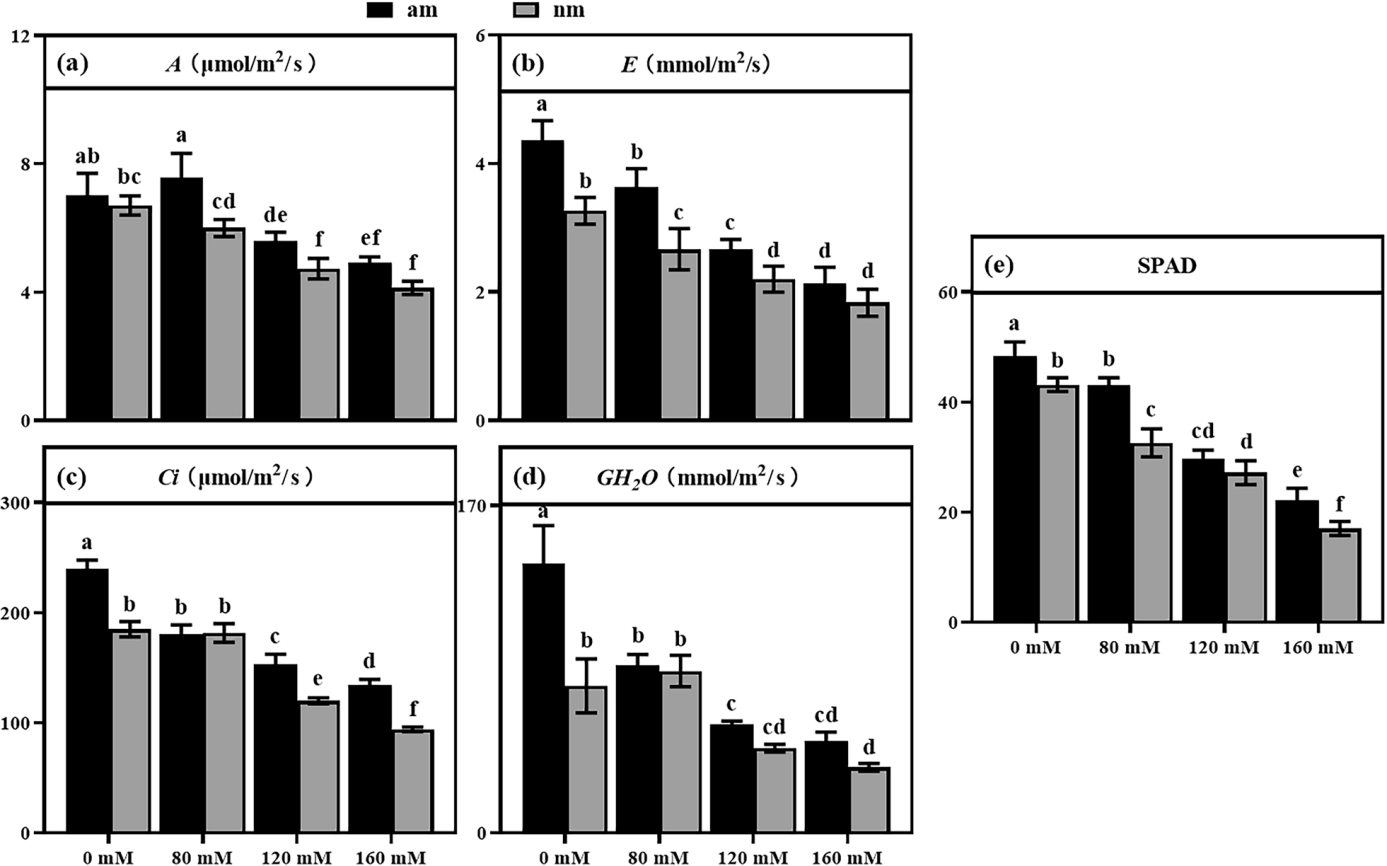
Photosynthetic gas exchange parameters and SPAD value of rice leaves between different treatment groups. Note: **a** *A*; **b** *E*; **c** *Ci*; **d** *GH*_*2*_*O*; **e** SPAD. The results are presented as the mean ± standard deviation of three replicates. Different letters indicate the difference between different treatment groups (*P* < 0.05)

Salt stress decreased the net photosynthetic rate of rice (Fig. 2 a, Table 1), and compared with the no salt treatment, the difference was significant (*P* < 0.05). The AMF compound inoculants can alleviate the effect of salt stress on net photosynthetic rate (*A*). At 80 mM and 120 mM salt concentrations, there was a significant difference between the am and nm treatment groups (*P* < 0.05), with the most significant difference at 80 mM salt concentration.

**Table 1:** Results of bidirectional variance analysis of effects of inoculum and salt concentration on rice seedling parameters

Parameter measured	Significance of sources of variation		
	Salt	AMF + PI + AR	Salt × AMF + PI + AR
Plant height	***	***	NS
Root length	***	***	*
Shoot Fresh weight	***	***	**
Root Fresh weight	***	***	**
Mycorrhizal	***	***	***
Spore number	***	***	***
Shoot K^+^ concentration	***	***	***
Root K^+^ concentration	***	***	*
Shoot Na^+^ concentration	***	***	NS
Root Na^+^ concentration	***	***	NS
Shoot Ca^2+^ concentration	***	***	NS
Root Ca^2+^ concentration	***	***	NS
Shoot K^+^/Na^+^ ratio	***	***	***
Root K^+^/Na^+^ ratio	***	***	NS
Shoot Ca^2+^/Na^+^ ratio	***	***	***
Root Ca^2+^/Na^+^ ratio	***	***	NS
Shoot/root Na^+^ ratio	*	NS	**
MDA	***	***	NS
SOD	***	***	*
POD	***	***	***
Gr	***	***	***
PAL	***	NS	NS
Pro	***	***	***
SPAD	***	***	**
*A*	***	***	NS
*E*	***	***	*
*Ci*	***	***	***
*GH*_*2*_*O*	***	***	***

*ns* not significant

*P < 0.05

**P < 0.01

***P < 0.001

Salt stress caused negative effects on the transpiration rate (*E*), stomatal conductance (*GH*_*2*_*O*), intercellular CO_2_ concentration (*Ci*)*,* and SPAD values of rice (Fig. 2 bcde). With increasing salt concentration these parameters all gradually decreased compared to those of non-salt stress. AMF compound inoculants can alleviate the damage of salt stress to rice photosynthesis, showing different results in different indicators. Under salt stress, there was a significant difference (*P* < 0.05) in *E* between am 0 and nm 0, am 80 and nm 80, and am 120 and nm 120. However, the difference in *E* between am 160 and nm 160 was not significant (*P* > 0.05). The effect on rice *GH*_*2*_*O* was only significantly different between the am and nm groups at 0 mM concentration (*P* < 0.05). In terms of *Ci*, the am treatment group showed a significant increase (*P* < 0.05) at 0 mM, 120 mM, and 160 mM concentrations compared to that of the nm group. Salt stress significantly reduced the SPAD value of chlorophyll parameters in rice (*P* < 0.05), and the AMF compound inoculants relieved the damage caused by the stress. There were significant differences from nm at 0 mM, 80 mM, and 160 mM salt concentrations (*P* < 0.05).

### Effects of AMF Compound Inoculants on Rice Biomass Under Salt Stress

**Fig. 3:**
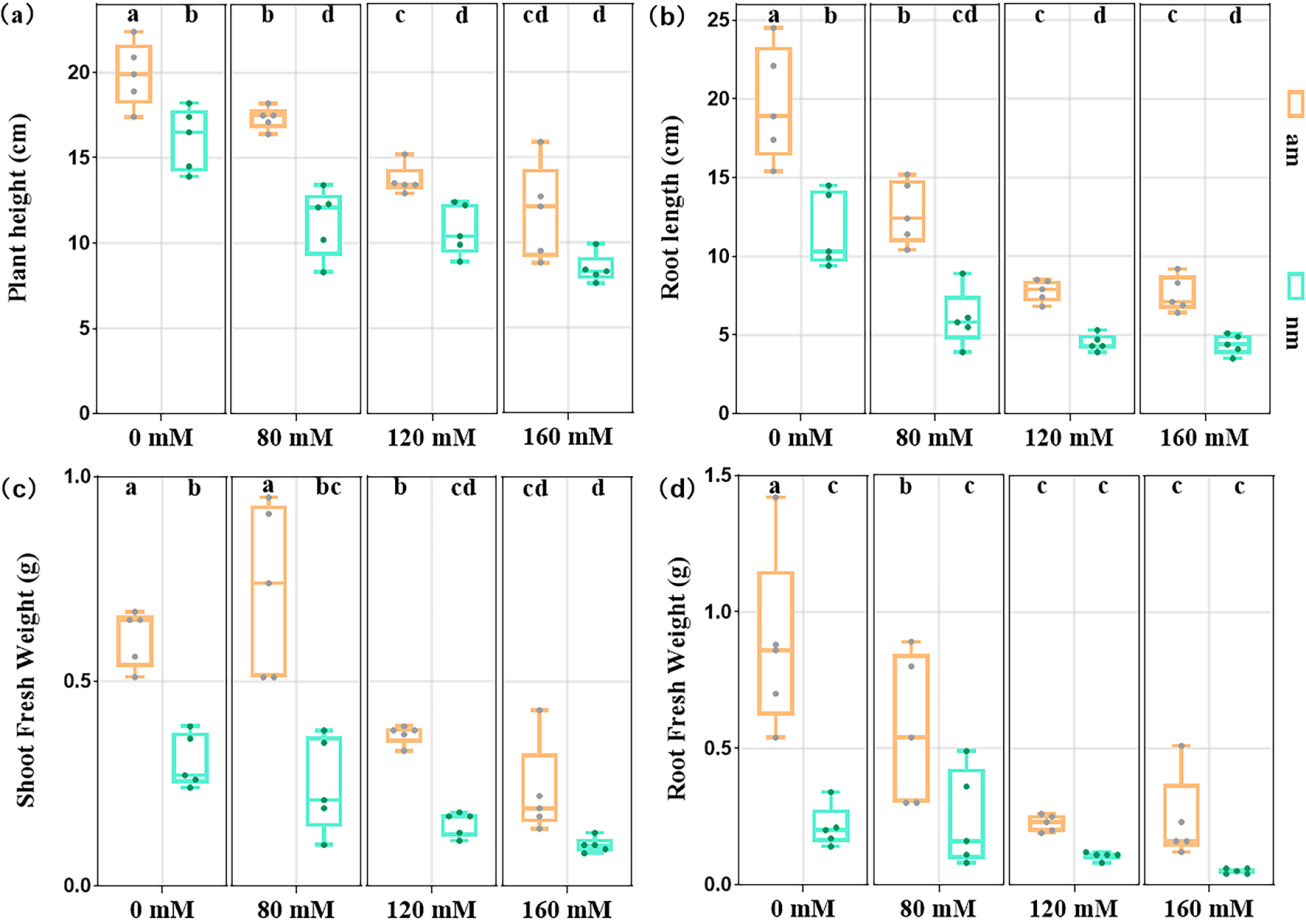
Growth parameters of *Oryza sativa* L*.* in different treatment groups. Note: **a** plant height; **b** root length; **c** shoot fresh weight; **d** root fresh weight. The results are presented as the mean ± standard deviation of three replicates. Different letters indicate the difference between different treatment groups (*P* < 0.05)

Measurement of rice growth indicators under different salt concentration treatments showed that co-inoculation with AMF compound inoculants significantly improved plant growth parameters (*P* < 0.05). However, the growth parameters of both plants inoculated with the complex agent and plants not inoculated with the complex agent were lower under stress conditions than those of inoculated and non-inoculated plants under stress conditions. AMF compound inoculants significantly reduced the toxic effects of salt stress. The most significant difference was between the am 80 and nm 80 treatment groups. The plant height and root length of rice increased by 53.98% and 111.58%, respectively. Damage to rice due to salt stress can be reduced by applying AMF compound inoculants. However, there is a limit to this reduction capacity, which tends to increase and then decrease with increasing salt concentration (Fig. 3, Table 1).

Low-salt stress (80 mM) increased the fresh weight of shoots in the am treatment group and the fresh weight of roots in the nm treatment group, but the difference was not significant (*P* > 0.05). However, with the increase in salt concentration, the fresh weight of shoots and roots of the am group and nm group decreased gradually. Compared with the nm treatment group, AMF compound inoculants could significantly increase the fresh weight of shoots and roots of rice under 80 mM and 120 mM salt stress (*P* < 0.05), but the fresh weight of roots was significantly lower at 120 mM salt concentration. In addition, there was no significant difference between the am and nm treatment groups as the salt concentration continued to increase, which indicated that the mitigation of rice growth and development under salt stress by AMF compound inoculants is limited.

### Effects of Salt Stress on the Contents of MDA, Osmoregulatory Substances, and Antioxidative Enzymes in Rice

**Fig. 4:**
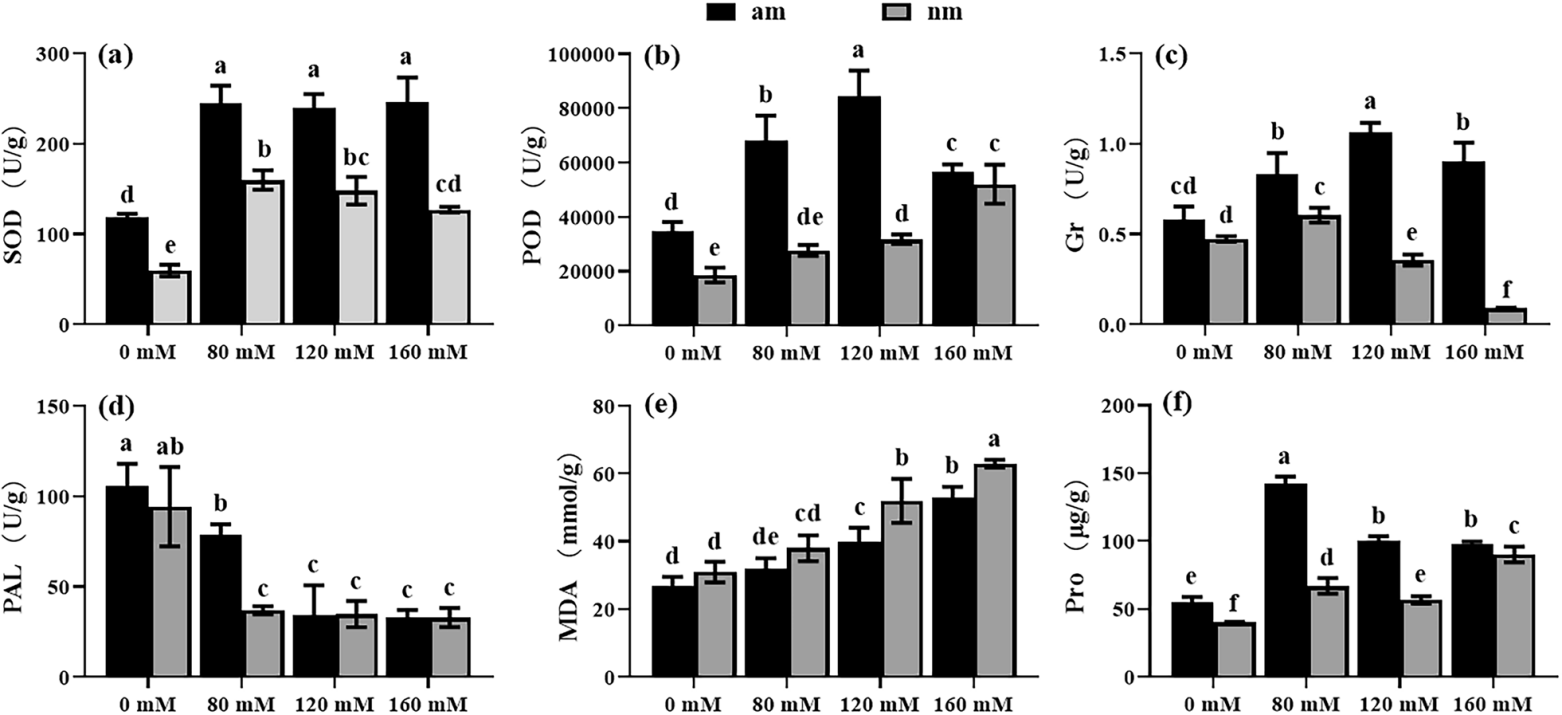
MDA contents, osmotic regulatory substances, and antioxidant enzyme activities in rice leaves. Note: **a** SOD; **b** POD; **c** Gr; **d** PAL; **e** MDA; **f** Pro. The results were the mean ± standard deviation of three replicates. Different letters indicate the difference between different treatment groups. (*P* < 0.05)

The malondialdehyde (MDA) content of rice leaves treated with different salt concentrations was measured (Fig. 4 e), and the results showed that salt stress led to an increase in MDA content, which significantly increased as the salt concentration increased (*P* < 0.05). The AMF compound inoculants can inhibit the increase in MDA content. The MDA content of the am0, am80, am120 and am160 treated groups was reduced by 12.91%, 15.79%, 23.07% and 15.87% in each group compared to that of the nm 0, nm 80, nm 120 and nm 160 treated groups, respectively. There was a significant effect of salt stress and AMF compound inoculants on the Pro content of rice leaves (Fig. 4 f, Table 1). Salt stress can induce a significant increase in Pro content in rice (*P* < 0.05), which increases with salt concentration. The AMF compound inoculants could further significantly increase the Pro content in rice and were significantly different from the nm treatment group at different salt concentrations (*P* < 0.05); the most significant difference in Pro occurred at 80 mM concentration, which was 2.12 times higher than that of the nm treatment group.

Salt stress significantly affected the content of SOD in rice leaves (Fig. 4 a, Table 1). Salt stress induced an increase in SOD in rice leaves and showed a trend of increasing first and then decreasing in the nm group. Compared with the nm treatment group, the am treatment group showed a significant increase in SOD content of rice leaves (*P* < 0.05), which were 1.98 times, 1.53 times, 1.61 times, and 1.93 times that of the nm treatment group, respectively. However, at concentrations of 80 mM, 120 mM, and 160 mM, there was no significant difference among the am group (*P* > 0.05).

Salt stress induced an increase in the POD content of rice leaves (Fig. 4 b, Table 1), and the AMF compound inoculants significantly increased the POD content of rice leaves in other groups except for the 160 mM group, which was 1.87, 2.46 and 2.66 times higher than that of the nm group, respectively. Low-salt stress increased the content of glutathione reductase (Gr) in rice leaves and inhibited the production of Gr in rice leaves as the stress level increased (Fig. 4 c), and the difference was significant (*P* < 0.05). The AMF compound inoculants increased the content of Gr in rice leaves, and there were significant differences at the levels of 80 mM, 120 mM, and 160 mM (*P* < 0.05). Among them, the am treatment group showed the highest increase at 160 mM, which was 10.10 times that of the nm group.

Salt stress significantly reduced the content of phenylalanine ammonia-lyase (PAL) in rice leaves (*P* < 0.05). AMF compound inoculants increase the PAL content in rice leaves under salt stress, but this increase is limited. PAL content was significantly different between the am and nm treatment groups at 80 mM salt concentration (*P* < 0.05) and not significantly different at 120 mM and 160 mM salt concentrations (*P* > 0.05) (Fig. 4 d).

### Ion Concentration and ion Homeostasis

**Fig. 5:**
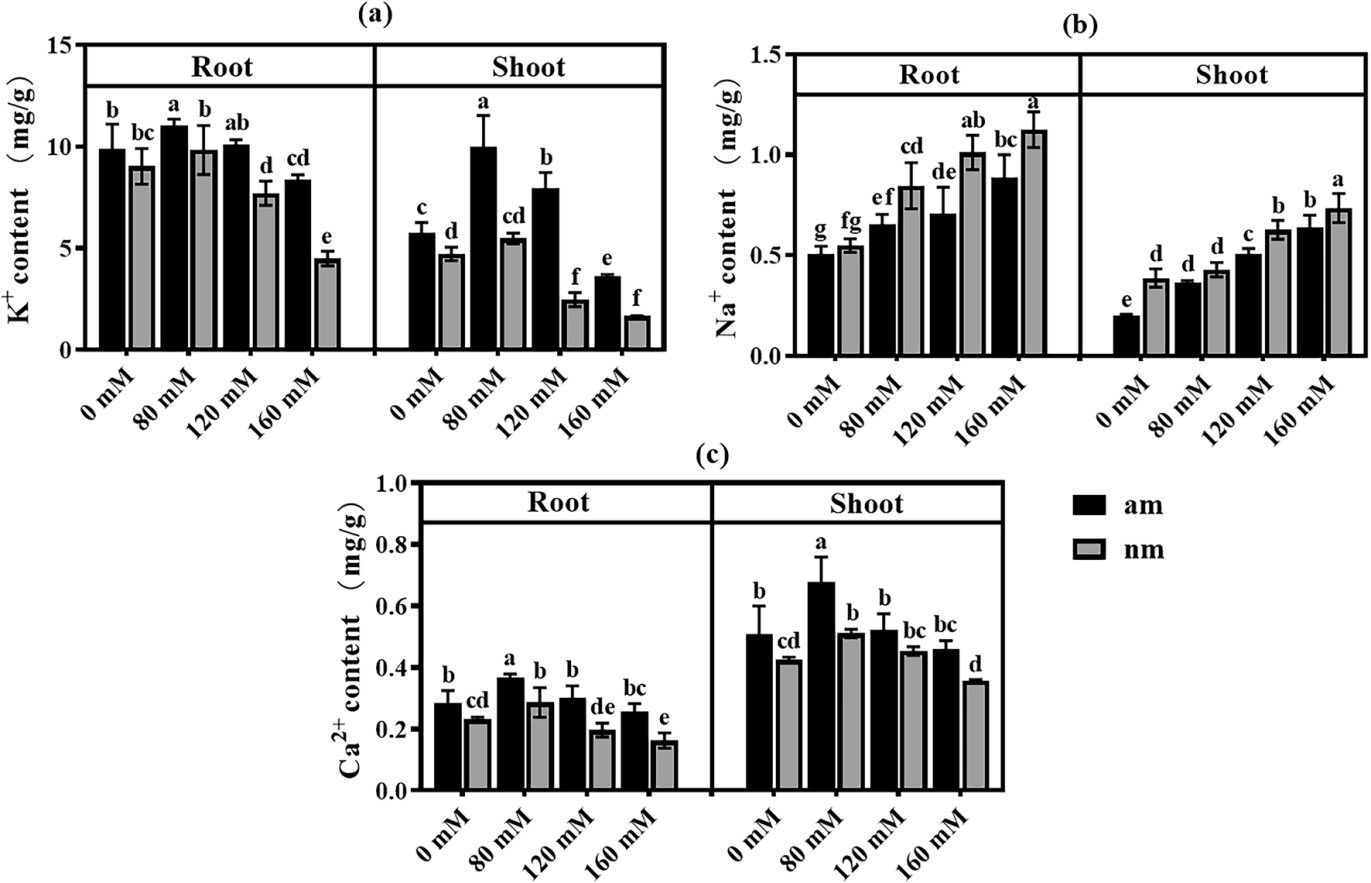
Aboveground and root ion contents of rice at different salt concentrations. Note: **a** K^+^ content; **b** Na^+^ content; **c** Ca.^2+^ content; the result is the mean ± standard deviation of three repeats. Different letters represent the differences between different treatment groups. (*P* < 0.05)

The contents of cations in the shoots and roots of rice seedlings were determined (Fig. 5, Table 1). It was found that more K^+^ accumulated in the roots than in the shoots under salt stress. Salt stress decreased the content of K^+^ in rice and decreased gradually with increasing salt concentration, which was significantly different from that of 0 mM (*P* < 0.05). However, the content of K^+^ increased slightly under the concentration of 80 mM salt, which was consistent in the shoots and roots of rice. The addition of AMF compound inoculants could increase the content of K^+^ in the shoots and roots of rice. There were significant differences in the K^+^ content in rice roots except for the 0 mM group (*P* < 0.05).

Salt stress increased the content of Na^+^ in the shoots and roots of rice. The content of Na^+^ gradually increased with increasing salt concentration. In the am and nm groups of rice, more Na^+^ was accumulated in the roots than shoots (*P* < 0.05) (Fig. 5 b). However, the Na^+^ content of both nonstressed plants inoculated with the compound inoculants and plants not inoculated with the compound inoculants was lower than that of inoculated plants and non-inoculated plants under stress conditions. Except for 80 mM, other concentrations in the aboveground parts of rice were significantly different from those of at 0 mM. AMF compound inoculants significantly reduced the content of Na^+^ in the shoots and roots of rice (*P* < 0.05). However, the difference in root Na^+^ content under salt-free conditions was not significant (*P* > 0.05).

Compared to the roots, the shoots of rice accumulated more Ca^2+^ (Fig. 5 C). Salt stress decreased the content of Ca^2+^ in rice, and decreased gradually with increasing salt concentration, which was significantly different from that of at 0 mM (*P* < 0.05). However, the content of Ca^2+^ increased slightly under the concentration of 80 mM salt, which was consistent in the shoots and roots of rice. The effect of salt stress on the Ca^2+^ content in the shoot of rice was not significant (*P* > 0.05). However, AMF compound inoculants could increase the content of Ca^2+^ in the shoots and roots of rice.

Salt stress negatively affected the shoot and root K^+^/Na^+^ ratios of rice with or without AMF compound inoculants (Tables 1, 2). However, the shoot ratio of rice increased slightly at the 80 mM concentration, and the difference was not significant (*P* > 0.05). Compared with the nonsalt condition, the shoot of rice under salt stress showed a significant difference above 120 mM (*P* < 0.05), while there was a significant difference in the root from 80 mM (*P* < 0.05). Inoculation with AMF compound inoculants at different salt concentrations significantly increased the ratio of K^+^/Na^+^ in the shoots and roots of rice (*P* < 0.05). The K^+^/Na^+^ of am 0, am 80, am 120 and am 160 under salt stress was 2.26-fold to 1.33-fold, 1.55-fold to 1.43-fold, 3.95-fold to 1.91-fold and 2.51-fold to 2.38-fold that of nm 0, nm 80, nm 120 and nm 160, respectively.

**Table 2:** Effects of salt and AMF compound inoculants on shoot K^+^/Na^+^ ratio, root K^+^/Na^+^ ratio, shoot Ca^2+^/Na^+^ ratio, root Ca^2+^/Na^+^ ratio in *Oryza sativa* L

NaCl(mM)	Treatment	Shoot K^+^/Na^+^	Root K^+^/Na^+^	Shoot Ca^2+^/Na^+^	Root Ca^2+^/Na^+^
0	nm	12.279 ± 0.671c	16.551 ± 2.217ab	1.110 ± 0.119c	0.423 ± 0.034b
	am	28.643 ± 2.807a	19.543 ± 0.952a	2.506 ± 0.425a	0.564 ± 0.074a
80	nm	12.831 ± 0.483bc	11.853 ± 2.699 cd	1.198 ± 0.067c	0.341 ± 0.053c
	am	27.253 ± 4.069a	16.977 ± 1.398ab	1.851 ± 0.205b	0.561 ± 0.037a
120	nm	3.954 ± 0.370de	7.683 ± 1.127e	0.728 ± 0.067de	0.197 ± 0.041d
	am	15.605 ± 0.828b	14.678 ± 2.761bc	1.032 ± 0.089 cd	0.432 ± 0.051b
160	nm	2.289 ± 0.206e	4.009 ± 0.267f	0.489 ± 0.047e	0.147 ± 0.031d
	am	5.752 ± 0.766d	9.522 ± 1.031de	0.733 ± 0.105de	0.292 ± 0.035c

Salt stress caused negative effects on the Ca^2+^/Na^+^ ratio of the shoots and roots of rice (Tables 1, 2). However, the shoot ratio of rice increased slightly under 80 mM, and the difference was not significant (*P* > 0.05). The Ca^2+^/Na^+^ of rice shoots in the am and nm groups produced significant differences from 0 mM at salt concentrations above 120 mM (*P* < 0.05). Ca^2+^/Na^+^ in the rice roots of the am and nm groups was significantly different from 0 mM at salt concentrations above 80 mM (*P* < 0.05). Compared to the nm group, the am group under salt stress showed shoot and root Ca^2+/^Na^+^ ratios of rice that were significantly increased (*P* < 0.05). The shoot and root Ca^2+^/Na^+^ in rice were 2.33 to 1.18, 2.12 to 1.65, 1.42 to 2.19, and 1.50 to 1.99 times higher in the am 0, am 80, am120, and am160 groups than in the nm 0, nm 80, nm120, and nm160 groups, respectively.

### Relative Gene Expression

#### Relative Expression of Salt Tolerance Genes in Rice

In this experiment, the am 80-treated and nm 80-treated groups with the most significant growth and physiological differences were selected for qRT‒PCR analysis. We selected eight rice salt tolerance-related genes, namely *OsQHB*, *OsPRX2*, *OsPRX4*, *OsPRX9*, *OsPRX72*, *OsPRX112*, *OsPRX-A2*, and *OsABSRP5*. We carried out qRT‒PCR verification of rice samples at 80 mM salt concentration. The relative expression of the *OsQHB*, *OsPRX2*, *OsPRX9*, and *OsPRX72* genes at the transcriptional level in the am-treated group was significantly different from that in the nm-treated group (*P* < 0.001) and they were 13.86-, 3.33-, 15.25-, 4.72-, 4.40-, and 1.95- fold that in the nm group, respectively. The relative expression of *OsQHB, OsPRX2, OsPRX9, OsPRX72, OsPRX112, OsPRX-A2*, and *OsABSRP5* transcript levels was significantly higher in the am-treated group than in the nm-treated group (*P* < 0.05).

#### Expression of Photosynthesis-Related Genes in Rice

The *OsHBP1b*, *Os10g21268*, *Os08g35420*, *Os05g25850*, *Os06g51150*, *Os10g38229*, *Os9g17740*, and *Os10g41780* genes can regulate the intensity of photosynthesis in rice. We carried out qRT‒PCR verification of rice samples at 80 mM salt concentration. No significant difference in *Os08g35420* expression was found between the am-treated and nm-treated groups under salt treatment (*P* > 0.05). The relative transcript levels of the *OsHBP1b, Os10g21268, Os05g25850, Os06g51150, Os10g38229, Os9g17740*, and *Os10g41780* genes were significantly higher in the am-treated group than in the nm-treated group (*P* < 0.001). The relative expression levels of the *Os10g21268, Os08g35420, Os05g25850, Os06g51150, Os10g38229, Os9g17740*, and *Os10g41780* genes in the am group were 22.56-, 57.03-, 17.02-, 28.47-, 3.82-, 10.29-, 9.95- and 5.11-fold, respectively.

#### Expression of Ion Transport-Related Genes in Rice

**Fig. 6:**
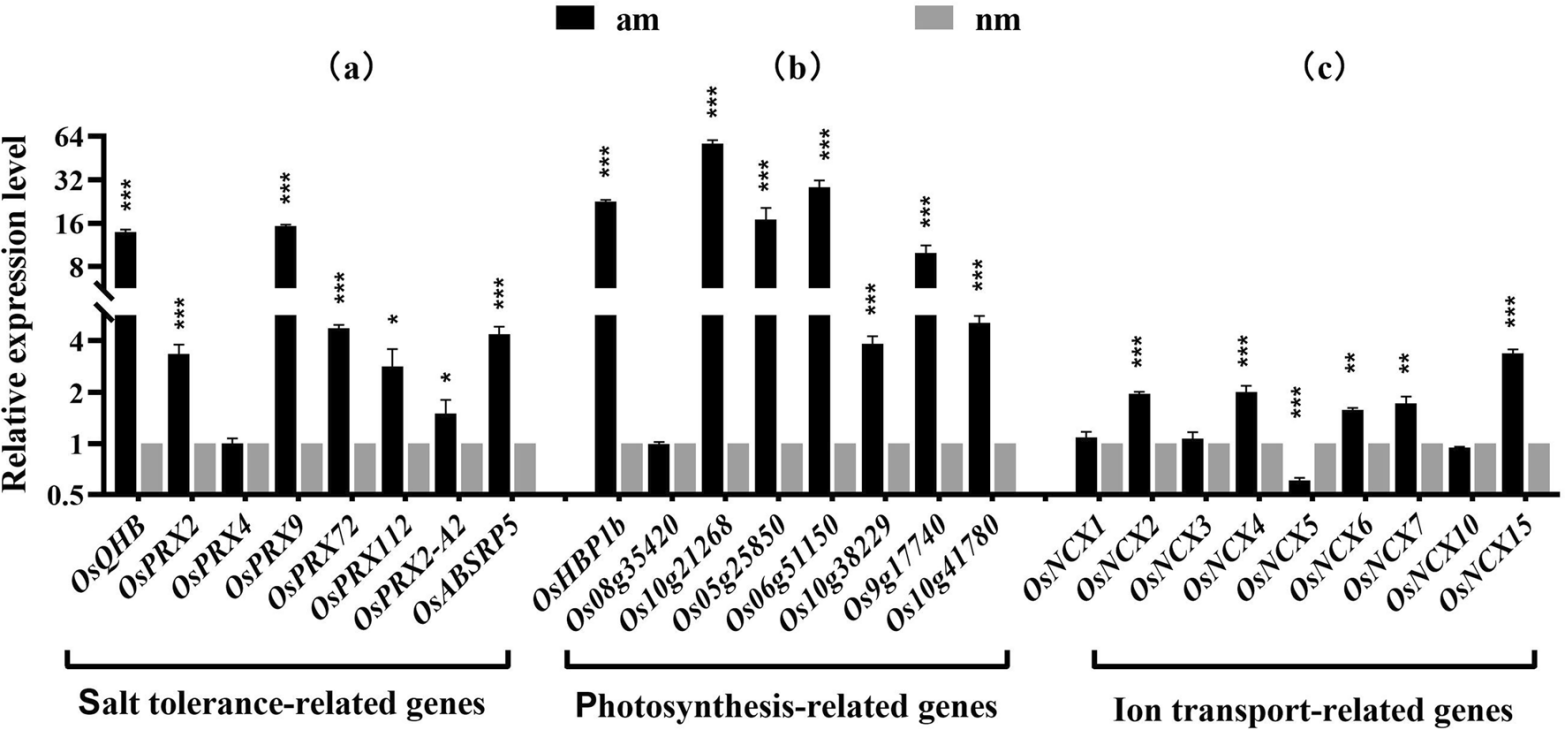
The relative gene expression of the am 80 treatment group and nm 80 treatment group. Note: **a** Relative expression of genes related to salt tolerance, **b** Relative expression of genes related to photosynthesis, **c** Relative expression of genes related to ion transport; * indicates the difference between different inoculation treatments when the same gene is treated (*P < 0.05; * *P < 0.01; ***P < 0.001)

In this experiment, the relative expression differences of genes related to cation exchange in rice were analysed. We selected nine genes, namely *OsNCX1*, *OsNCX2*, *OsNCX3*, *OsNCX4*, *OsNCX5*, *OsNCX6*, *OsNCX7*, *OsNCX10* and *OsNCX15*. We carried out qRT‒PCR verification of rice samples at 80 mM salt concentration. There no significant difference in the relative expression of *OsNCX1*, *OsNCX3*, and *OsNCX5* at the transcriptional level between the am-treated group and the nm-treated group (*P* > 0.05). There was a highly significant difference in the relative expression of *OsNCX2, OsNCX4, OsNCX6, OsNCX7*, and *OsNCX15* transcript levels between the am-treated and nm-treated groups (*P* < 0.001). The relative expression levels of *OsNCX2, OsNCX4, OsNCX6, OsNCX7*, and *OsNCX15* transcript levels in the am group were 1.97-, 2.01-, 1.57-, 1.72-, and 3.37-fold of those in the nm group (Fig. 6).

### Principal Component Analysis (PCA)

**Fig. 7:**
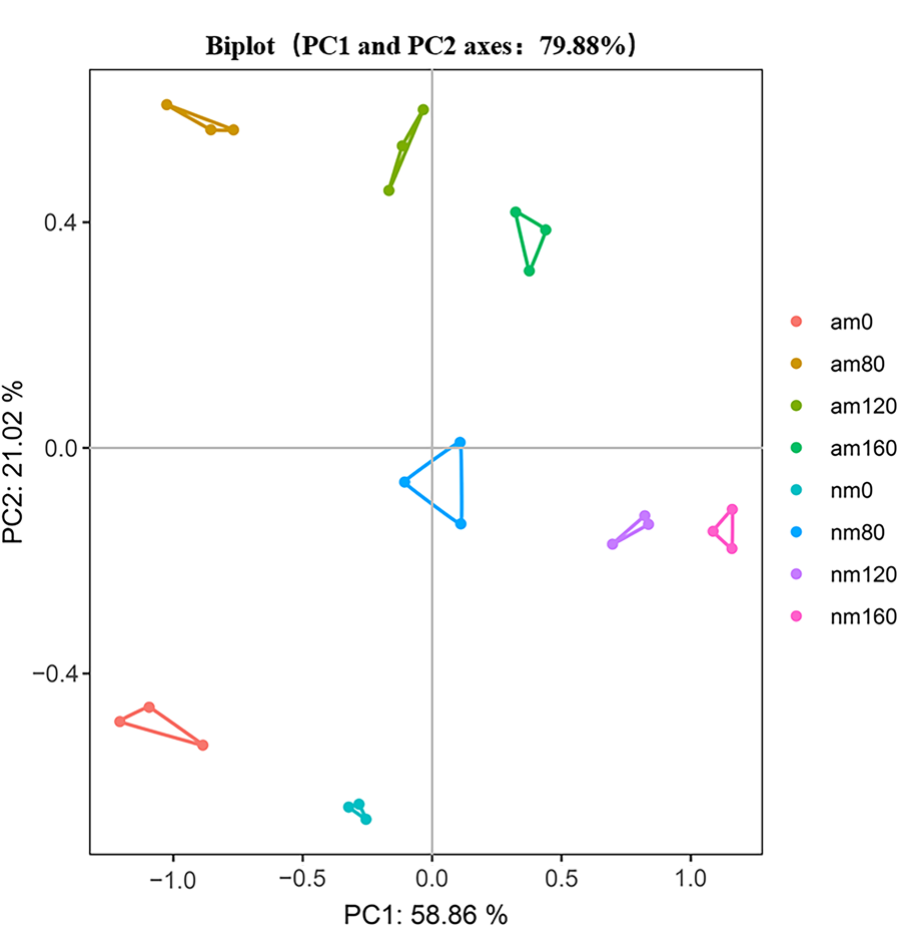
Principal component analysis (PCA) in *Oryza sativa* L. under salt and AMF compound inoculant treatments. Note: nm: non-mycorrhizal; am: Fm + Pi + Ar.0: 0 mM NaCl; 80: 80 mM NaCl; 120: 120 mM NaCl; 160: 160 mM NaCl

Principal component analysis was carried out on the data of rice biomass, mineral ion absorption, homeostasis, ROS scavenging enzymes, light, and parameters. The X-axis and Y-axis denote the first (PC1) and second (PC2) principal components, respectively (Fig. 7), with a total PCA explanation of approximately 79.88%. The results of this study show that there is a clear distinction between different samples. The differences between salinity levels were distinguished by PC1, while PC2 tended to distinguish the mycorrhizal inoculum status of the samples. In the scatter plot of PC1 and PC2, a smaller distance between the samples indicates similarity. The am treatment group and the nm treatment group are obviously separated. Under the same salt concentration, the difference between the am 80 group and nm 80 group was the most significant. There was the most significant difference between the am 0 group and nm 160 group under different salt concentrations.

## Discussion

### Effect of Inoculation with AMF Compound Inoculants on the Infection and Biomass of Rice Seedlings Under Sodium Chloride Stress

*Agrobacterium rhizogenes* infecting plants can induce the root system to differentiate many fast-growing hairy roots and increase the root area and biomass (Wang et al. 2020a, b). It has been shown that *Agrobacterium* can act as a mycorrhizal auxotroph to directly promote AMF spore germination, mycelial growth and colonization of plant roots by AMF and Indian pear-shaped spores (Frey et al. 2007). Previous studies found that the mycorrhizal infection rate of the AMF complex was significantly higher than that of other treatments (Zhang et al. 2022). Additionally, AMF can promote plants to release root secretions. In turn, root secretions are the main source of nutrients for plant rhizosphere bacteria, which promote the infection of root-emitting *Agrobacterium* (Paul et al. 2011). *Piriformospora indica* ecological functions are similar to those of AMF and can act synergistically. The combined application of AMF and Pi promotes the growth and photosynthetic efficiency of plant seedlings. In the present study, the AMF compound inoculants increased the biomass of rice seedlings under salt stress and positively affected the light and gas exchange parameters and proline content of rice seedlings. This is consistent with the findings of Moreira et al. (2015). However, whether Ar can be used as an active agent to improve AMF infection and the compound infection mechanism of AMF and Pi need to be further investigated.

Rice is most sensitive to salt stress at the seedling stages. The salt tolerance of rice seedlings is an important indicator to for evaluating the salt tolerance of rice during the whole reproductive period (Das et al. 2019; Singh et al. 2015). NaCl stress may negatively affect fungal growth, spore formation, and infection (Peng 2019; Wang et al. 2020a, b). This study found that rice seedlings can establish a good symbiosis with AMF compound inoculants. In contrast, at a salt concentration of 80 mM, both mycorrhizal infection rates were elevated, but the spore number in rhizosphere soil was significantly reduced. This may be because low salt stress promoted the release of AMF secretions and the formation of mycorrhizal symbiosis in the rice root system. However, the spore reproduction capacity of AMF was reduced due to the inhibition of salt stress. The mycorrhizal infection rate decreased significantly as the salt concentrations continued to increase, which may be due to the negative effect of high salt concentration on mycelial growth and sporulation (Zhou et al. 2022). Compared to the nm-treated group, the am-treated group did not show significant improvements in some indices, such as *A, E, GH*_*2*_*O*, root length, fresh weight, and POD, under high concentrations of salt stress. This may indicate that there is some positive correlation between the infection rate of AMF compound inoculants on rice and the improvement in salt tolerance in rice. We speculate that there is a greater potential for effective infection of AMF compound inoculants in alleviating salt stress in rice plants.

Salt stress affects many metabolic processes in plants, resulting in slow growth and development (Lutts et al. 1995). Many studies have shown that mycorrhizal plants can mitigate the adverse effects of salt stress by activating antioxidant enzyme systems(Shrivastava et al. 2015; Kakar et al. 2019). Mycorrhizal plants can absorb more mineral nutrients and water from the soil, increasing the osmotic potential of the soil solution and allowing the plants to grow and develop properly. Evelin et al. (2012) found that the biomass of mycorrhizal *Trigonella foenum-graecum* increased significantly under different concentrations of salt stress treatment. Similar results were obtained in the present study. Under salt stress conditions, the plant height, root length, root fresh weight, and aboveground fresh weight of mycorrhizal rice seedlings were significantly increased. Mycorrhizal plants may attenuate the toxic effects of salt stress on plants by maintaining high biomass (Parvin et al. 2020). In this study, salt stress significantly reduced the biomass of rice seedlings, while inoculation with the AMF compound inoculants improved rice growth and physiological status. The decrease in mycorrhizalized rice biomass was smaller than that of the control treatment, which facilitated the better adaptation of rice to salt stress.

### Effect of Inoculating AMF Compound Inoculants on Photosynthetic gas Exchange Parameters and SPAD Values of Rice Under NaCl Stress

Salt stress reduces the photosynthetic capacity of plants mainly by destroying photosynthetic organs and reducing photosynthetic pigment content. A chloroplast is an important site of photosynthesis, and its chlorophyll content is among the main indices of plant photosynthetic capacity(Pitman et al. 2002). The SPAD value represents the relative content of chlorophyll in plants. The results showed that salt stress decreased the chlorophyll content. AMF compound inoculants can increase the SPAD value, thus promoting the photosynthesis of rice seedlings.

Photosynthesis is an important physiological process that affects plant growth. Previous studies have shown that inoculation with AMF can improve the intensity of photosynthesis in plants(Frosi et al. 2018). The results of the study also showed that the photosynthetic capacity of leaves of mycorrhizalized beech *Zelkova serrata* (Thunb.) Makino was higher than that of non-mycorrhizalized *Zelkova serrata* (Thunb.) Makino at a salt concentration of 100 mM(Peng et al.2020; Wang et al. 2019). In this experiment, we found that the *E, GH*_*2*_*O*, *A,* and *Ci* of rice leaves in the am treatment group were higher than those in the nm treatment group. Mycorrhizal rice may improve photosynthetic capacity by increasing photosynthetic gas exchange capacity and water absorption, to reduce the toxicity caused by salt stress. This is consistent with the findings of Chang et al. (2018). The results showed that the inoculation of AMF compound inoculants improved the water use and light energy efficiency of rice under salt stress, and effectively improved the salt tolerance of rice seedlings.

### Effect of AMF Compound Inoculants on the Antioxidant Enzyme System, MDA, and Pro Content of Rice

Plants mitigate damage from salt stress by secreting antioxidant enzymes and synthesizing osmoregulatory substances (Garcia-Garrido and Ocampo 2002). The experimental results showed that under salt stress conditions, the MDA content of rice leaves increased under increasing salt stress and was significantly higher than that of non-salt stress treatments. This indicates that salt stress induces increased levels of intracellular ROS in rice plants, which subsequently causes oxidative damage to cell membrane structures. Under salt stress, the MDA content of mycorrhizal rice seedlings was significantly lower than that of non-mycorrhizal rice seedlings. The results showed that inoculation with AMF compound inoculants could reduce the degree of membrane lipid peroxidation in rice seedlings under salt stress. Chen et al. (2022) found that inoculation with AMF could significantly increase the activity of antioxidant enzymes in *Alhagi sparsifolia* seedlings under salt stress. It also promoted the accumulation of osmoregulatory substances in seedlings of *Alhagi sparsifolia*, and significantly reduced the content of MDA. Combined with the results of previous studies, it was further clarified that AMF compound inoculants could improve the resistance of plants to salt stress.

Excess salt causes a decrease in soil osmotic potential, preventing plants from absorbing water from the soil and causing physiological drought in plants. A large amount of osmoregulatory substances such as free proline, are produced and accumulated in plant cells; these substances can reduce the intracellular osmotic potential and ensure normal water uptake and utilization by plants. Plants utilize this strategy to cope with physiological drought caused by salt stress (Ahmed et al. 2020). This physiological change ensures normal water uptake and utilization by plants, thus alleviating the physiological drought caused by salt stress. In addition, it has been suggested that proline in plants also plays a variety of important functions, such as scavenging ROS, and stabilizing proteins and cell membrane structures(Deinlein et al. 2014). In the present experiment, we found that the proline content of rice seedlings significantly increased under salt stress at 80 mM, 120 mM, and 160 mM. Inoculation with AMF compound inoculants further increased the proline content in rice seedlings. The results showed that inoculation with AMF compound inoculants could induce the accumulation of proline in rice under salt stress, reduce the cellular osmotic potential and improve the salt tolerance of rice seedlings. Similar results were obtained in the salt tolerance test of mycorrhizalized cucumber conducted by Han (2012).

Under salt stress, plants produce more harmful oxygen radicals, which interfere with the normal metabolic activities of the plant body and inhibit plant growth and development (Didi et al. 2022). In plants, SOD catalyses the conversion from O^2−^ to O_2_ and H_2_O_2_. Then POD and others reduce the damage caused by salt stress by catalysing H_2_O_2_ to H_2_O and O_2_ (Wang et al. 2023). It was previously found that AMF can effectively reduce the level of membrane lipid peroxidation and enhance the tolerance of plants to weak light and salt stress by promoting the growth and antioxidant enzyme activity of *Cucumis melo* L. under weak light and salt stress (Xu 2017). The results of Cao showed that inoculation of AMF under salt stress could effectively reduce the level of cell membrane lipid peroxidation by promoting the accumulation of osmotic regulators and increasing the activity of antioxidant enzymes in *Asparagus officinalis* L. This alleviates the damage of salt stress to the plant (Cao 2017). In this experiment, salt stress increased the antioxidant enzyme activities in rice. However, under high salt stress, some antioxidant enzyme activities decreased. This indicates that the resistance of rice to salt stress is limited. The activity of antioxidant enzymes in rice was significantly increased by inoculation with AMF compound inoculants at all concentrations. It is suggested that AMF compound inoculants can improve the salt tolerance of rice by increasing the activities of antioxidant enzymes. This result is similar to those of previous studies, indicating that AMF compound inoculants can improve plant salt tolerance by increasing the activities of antioxidant enzymes in plants.

### Ion Content and Dynamic Equilibrium

The sensitivity of plants to salt mainly depends on the absorption, accumulation, and distribution of Na^+^. Salt stress increased the content of Na^+^ in the roots and shoots of rice seedlings, and gradually increased with the increasing salt stress. In this study, inoculation with AMF compound inoculants effectively reduced the content of Na^+^ in the shoots and roots of rice. The content of Na^+^ in the shoots was lower than that in the roots. The results showed that inoculation with AMF compound inoculants inhibited the transport of Na^+^ from rice roots to leaves. This may be a method for mycorrhizal plants to inhibit the accumulation of toxic ions in photosynthetic tissues (Porcel et al. 2016).

Potassium is one of many mineral elements necessary for plant growth. In the present study, salt stress showed the same pattern for the accumulation of K^+^ in all parts of rice. Salt stress (80 mM salt stress could increase) increased the content of K^+^ in the shoots and roots of rice. However, an excess of salt reduces the content of K^+^. Compared with the nm group, inoculation with AMF compound inoculants increased the content of K^+^ in rice and maintained a higher level of K^+^/Na^+^. The results showed that AMF compound inoculants promoted the accumulation of K^+^ in rice. Mycorrhizal plants regulate the ion homeostasis of plants. This is consistent with the results obtained by Wang et al. (2022a, b, c).

In plants, Ca^2+^ is involved in many important biological processes, such as protecting the structural and functional integrity of plant cell membranes, stabilizing cell wall structure, and regulating ion transport and selection. In this study, 80 mM salt stress slightly increased the content of Ca^2+^ in rice plants. However, with the increase in salt concentration, the content of Ca^2+^ decreased, and the content of Ca^2+^/Na^+^ decreased with salt stress. This suggests that salt stress inhibits Ca^2+^ transport by plants. Inoculation with AMF compound inoculants can increase the Ca^2+^ content in rice shoots and roots, which can promote Ca^2+^ uptake by rice and reduce ion loss caused by salt stress.

Inoculation with AMF compound inoculants increased the contents of K^+^ and Ca^2+^ in rice plants under both salt-stressed and nonsalt-stressed conditions. This may occur because, mycorrhizal symbionts increase root length and root area through the action of hyphae and hairy roots, which effectively improves the ability of plants to absorb mineral nutrients. On the other hand, K^+^ and Ca^2+^, as osmotic substances, can be selectively absorbed by mycorrhizal symbionts and transported to plant organs and tissues to prevent plants from absorbing more Na^+^ (Hammer et al. 2011). These results suggest that inoculation with AMF compound inoculants can regulate ion homeostasis in plants and help rice adapt to different concentrations of salt stress.

### Relative Gene Expression

AMF contains multiple SOD-encoding genes and induces an increase in plant SOD isoforms (Cu–Zn SOD, Mn-SOD and Fe-SOD). The symbiotic mechanism of AMF increases the content and concentration of antioxidant enzymes in plants and upregulates the expression of related genes (Evelin et al. 2012). AMF induces upregulated expression of ASA-GSH (ascorbate–glutathione) cycle-related enzymes in the host plant, maintains high antioxidant content, and prevents free radicals and membrane fatty acid interactions, thus increasing membrane stability and preventing protein denaturation (Hashem et al.^2015^). Chang et al. (2023) investigated the effect of salt stress on mycorrhizal *Elaeagnus angustifolia* L. by LFQ proteomics (label-free quantification). Mycorrhizal symbiosis helps the host plant *Elaeagnus angustifolia* L. respond positively to salt stress and improve its salt tolerance by regulating the activity of some key proteins related to amino acid metabolism, lipid metabolism and glutathione metabolism in root tissues. AMF can increase the content and activity of antioxidant enzymes by regulating the expression of plant-related genes and can regulate the activity of certain metabolic key enzymes in plants to improve salt tolerance in host plants.

Excessive accumulation of ROS can cause oxidative damage to cells. To alleviate this damage, plants have evolved a defense system for scavenging ROS to increase the activity of ROS scavenging enzymes and enhance salt tolerance. Genes such as *PRX* and *APX* can regulate the activity of ROS scavengers (Liu et al. 2021b; Savina et al. 2020). Zhou et al. (2022) found that overexpression of the *OsQHB* gene enhanced salt tolerance in rice. Multiple peroxidase (*PRX*) genes are regulated by *OsQHB*, resulting in higher ROS scavenging enzyme activity and lower MDA accumulation in rice. In this study, *OsQHB* and its related *PRX* genes were analysed by qRT‒PCR. The results showed that mycorrhizal rice exhibited higher gene expression under salt stress at 80 mM. This is consistent with the results of the study on the activity of ROS-scavenging enzymes mentioned in this paper. Mycorrhizal symbionts may induce overexpression of *OsQHB* and regulate related *PRX* genes, thus increasing the activity of ROS scavenging enzymes. It is helpful to improve the salt tolerance of rice, but the mechanism of overexpression behind the related genes induced by mycorrhizal symbionts is unclear.

Salt stress has a serious negative impact on plant productivity, and plant stress resistance and yield are regulated by transcription factors (Mbodj et al. 2018). In addition, activating related transcription factors can greatly affect the stress resistance of plants. It was previously found that the *OsHBP1b* gene can regulate and maintain high chlorophyll concentrations in plants (Lakra et al. 2015). The overexpression of this gene can improve rice growth and photosynthesis activity. Overexpression of *OsHBP1b* in rice signifies its own role in stress management by minimizing the level of ROS, increasing antioxidant enzyme activity, maintaining organelle structure, increasing photosynthesis and modulating the level of photosynthesis and stress-related transcripts. In addition, the *OsHBP1b* gene enhanced stress tolerance in rice and was associated with the ABA (abscisic acid) signalling pathway (Das et al. 2019). In this experiment, the *OsHBP1b* gene was analysed by qRT‒PCR. The results showed that the expression of *OsHBP1b* was higher in mycorrhizal rice under 80 mM salt stress. It was also verified that mycorrhizal rice exhibited stronger photosynthetic gas exchange parameters and higher SPAD values under salt stress.

Salt tolerance in rice is a quantitative trait controlled by multiple genes. Salt stress can activate some genes related to salt tolerance in rice to maintain a high level of K^+^/Na^+^ and maintain the integrity of the cell membrane (Wang et al. 2020a, b). The dynamic balance of Na^+^, K^+^, and Ca^2+^ is the key factor affecting plant growth and development. Singh studies have shown that *NCX* protein can change the level of intracellular ions, especially Ca^2+^. Differential expression of *NCX* genes plays an important role in the physiological processes of rice (Singh et al. 2015). In this experiment, the *OsNCX* gene was analysed by qRT‒PCR. The results showed that the expression of the *OsNCX* gene was higher in the am 80 treated group under salt stress at 80 mM. It was also verified that mycorrhizal rice exhibited higher K^+^ concentrations and lower Na^+^ concentrations, maintained a higher K^+^/Na^+^ and could adapt to salt stress.

This study shows that mycorrhizal symbionts play an important role in improving the salt tolerance of rice. This study provides a theoretical basis for further revealing the regulatory mechanism of mycorrhizal symbionts on rice growth under salt stress. The salt tolerance genes, photosynthesis-related genes, and cationic regulatory genes of rice seedlings were verified by qRT‒PCR. However, the corresponding metabolic pathways and upstream and downstream related regulatory genes are still unclear and need to be further studied.

## Conclusion

AMF compound inoculants significantly alleviated the damage caused by different concentrations of salt stress. The effect is the most significant at a salt concentration of 80 mM. At 80 mM salt, the AMF compound inoculants significantly increased the activities of antioxidant enzymes in rice leaves. AMF compound inoculants increased the content of osmotic regulatory substances and the contents of K^+^/Na^+^ and Ca^2+^/Na^+^. AMF compound inoculants reduced the damage to plasma membrane peroxidation caused by salt stress and increased the biomass of rice seedlings. The relative expression of the *OsQHB*, *OsHBP1b,* and *OsNCX* genes increased significantly. This increase provides favorable conditions for rice to survive in salinized soil. AMF compound inoculants have the potential to improve the production of rice in salinized soil and have certain application prospects.

## Materials and Methods

### Material Preparation

Rice (*Oryza sativa* L.) seeds were selected from Longjing 31, a salt-intolerant variety mainly planted in Northeast China, and were purchased from the Jiansanjiang Farm Management Bureau, Heilongjiang Province.

The following strains of AMF compound inoculants were included: (1) *Funneliformis mosseae* (Fm, isolate number: CGMCC No. 3012): Obtained by pot expansion using sorghum as host (spore number ~ 39/g); (2) *Piriformospora indica* (Pi): *P. indica* fungal liquid (Spore content 2.5 × 10^5^ CFU/mL); (3) *Agrobacterium rhizogenes* (Ar): (Bacteria liquid 3.0 × 10^5^ CFU/mL). The above strains were preserved by the Restoration Ecology Research Laboratory of Hei-Longjing University.

**Fig. 8:**
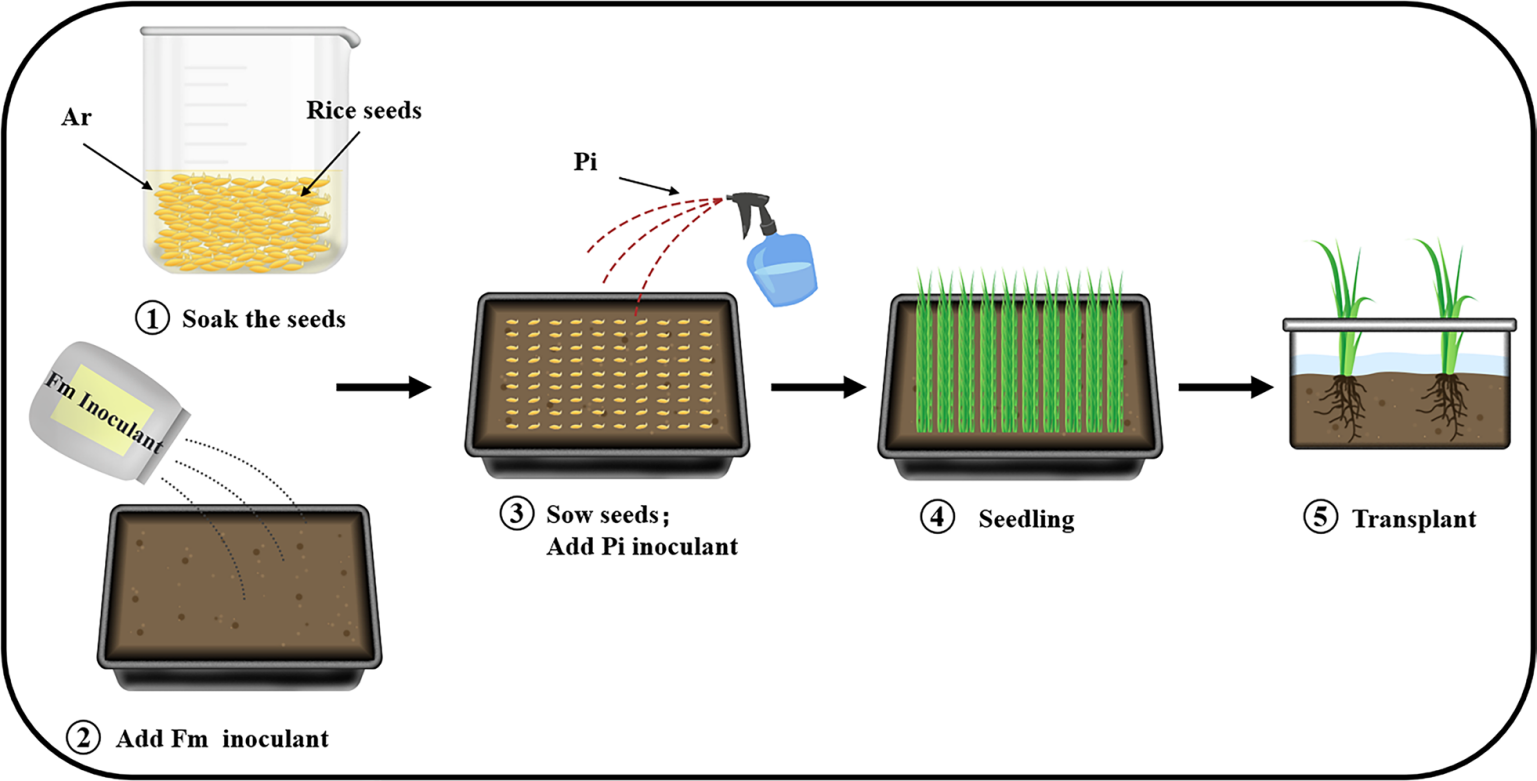
Nursery process of AMF compound inoculants

The AMF compound inoculants were inoculated as follows: the Fm inoculants (5%, w/w) were mixed with the rice seedling soil and used as the rice seedling substrate; the germinated rice seeds were soaked in the *A. rhizogenes* bacteria liquid with a rice seed to bacterial liquid ratio of 1:1 (w/v), and a soaking time is 8–16 h; the soaked seeds were sown in the seedling substrate; and after diluting 100 mL of Pi fungal liquid into 500 mL of bacteria liquid with sterile water, the solution was sprayed evenly on the surface of the seeds(one seedling tray needed 500 mL of Pi fungal liquid, i.e., 15 L/hm^2^).Thus the inoculation of the AMF compound inoculants was completed (Fig. 8).

### Experimental Design and Methods

#### Experimental Design

The pot experiment began in June 2022; rice seeds with full grains were selected, sterilized with 5% NaClO for 10 min, and washed five times with sterile water. The seeds were placed into a constant temperature incubator at 30 °C to accelerate germination, and when the germination rate reached more than 80%, we performed the above inoculation process with AMF compound inoculants, cultivated them in seedling trays (length: 45 cm, width: 30 cm, height: 5 cm) and covered them with plastic film to maintain high humidity. On the 20th day of growth, rice seedlings were transplanted into rectangular plastic pots (upper aperture 33 cm × 16 cm; lower aperture 25 cm × 10 cm; height 14 cm) to simulate paddy culture; the pots were lined with double waterproof plastic bags to prevent water loss. Each pot was filled with 3 kg of sterilized soil (the soil was autoclaved at 121 °C for 120 min), exogenous salt was added to the soil with NaCl (analytically pure) solution, and 2.5 L of NaCl solution with different concentrations was added to each pot.

The experimental design included the following experimental factors: AMF compound inoculants and NaCl stress. The following levels of treatment were used for the AMF compound inoculants treatment: AMF compound inoculants inoculum and sterilized inoculum, and four levels of treatment were used for NaCl treatment (0 mM, 80 mM, 120 mM, 160 mM NaCl). In this experiment, a total of eight treatment combinations were used, including nm 0 (sterilized inoculum + 0 mM NaCl), am 0 (AMF compound inoculants inoculum + 0 mM NaCl), nm 80 (sterilized inoculum + 80 mM NaCl), am 80 (AMF compound inoculants inoculum + 80 mM NaCl), nm 120 (sterilized inoculum + 120 mM NaCl), am 120 (AMF compound inoculants inoculum + 120 mM NaCl), and nm 160 (sterilized inoculum + 160 mM NaCl), am 160 (AMF compound inoculants inoculum + 160 mM NaCl). Six replicates of each treatment were randomly arranged with 5 seedlings per pot, for a total of 48 pots.

#### Determination of the Rice Mycorrhizal Infection Rate and Spore Number in Rhizosphere Soil

The 42 days of rice seedling transplanting coincided with the tillering stage of the rice sample. This period is the most sensitive period of salt stress for rice seedlings and can reflect the salt tolerance of rice throughout its life cycle. And in this period, rice seedlings under high concentrations of salt stress showed signs of death, so it was representative. Therefore, we collected samples at this time for measurements. Fifty to one hundred root segments in each treatment were randomly selected, stained with the Trypan blue staining method, transparent, stained, decolorized, sliced, and observed under a 10 × 40 microscope. The mycorrhizal infection rate was calculated as follows (Feng and Song 2020).

$$ {\text{root}}\;{\text{colonization}}\;(\% ) = \frac{{{\text{Number}}\;{\text{of}}\;{\text{arbuscular}}\;{\text{mycorrhiza}} - {\text{positive}}\;{\text{segments}}}}{{{\text{Total}}\;{\text{number}}\;{\text{of}}\;{\text{segments}}\;{\text{studied}}}} \times 100\% $$

Soil spore counts were performed by the wet sieve method (Sun et al. 2022). Five grams of air-dried soil was weighed and placed on a soil sieve with a pore size of 0.5–0.0385 mm. The washing solution was collected and filtered in a funnel, the filter paper loaded with spores were placed on a clean Petri dish, and the spores were observed and counted with a stereomicroscope.

#### Determination of Photosynthetic Gas Exchange Parameters and SPAD Values of Rice

Photosynthetic gas exchange parameters were determined using an LI-6400 photosynthesis instrument (LI-6400XT, LI-COR Corporate, Lincoln, Nebraska USA, USA). On August 11, 2022 (42 days of rice seedling cultivation), from 9:00 to 11:00 a.m., plants with consistent growth were selected in each treatment to measure the relevant indices, and each treatment was measured three times. The indicators measured include net photosynthetic rate (*A*), stomatal conductance (*GH*_*2*_*O*), intercellular CO_2_ concentration (*Ci*), and transpiration rate (*E*). The SPAD value of rice leaves was measured with a chlorophyll analyser (FK-YL01, Shandong Fangke Instrument Co., Ltd., Weifang, China).

#### Determination of Rice Biomass

On the 42nd day of rice growth, five rice plants were taken from each treatment. First, the aboveground and root parts of the plant, were separated, the roots were cleaned with tap water and washed it with deionized water three times, and the surface water was dried with filter paper. Finally, the plant height and shoot and root fresh weight were measured.

#### Determination of MDA Contents, Osmotic Regulatory Substances, and Antioxidant Enzyme Activities of Rice

The Superoxide Dismutase Kit (Solarbio Life Sciences, BC0175), Peroxidase Kit (Solarbio Life Sciences, BC0095), Glutathione Reductase Kit (Solarbio Life Sciences, BC1165), Malondialdehyde Kit (Solarbio Life Sciences, BC0025), Proline Kit (Solarbio Life Sciences, BC0295), and Phenylalnine Ammonialyase (PAL) Activity Assay Kit (Solarbio Life Sciences, BC0215) were used to determine the enzyme activity of rice according to the manufacturer’s instructions.

#### Determination of Ion Concentration

Rice samples were dried at 105 °C for 30 min and then dried at 70 °C until constant weight. Plant sample parts were ground and sieved (80 mesh). The sample was placed in a digestion tube, 10 mL nitric acid was added and the samples were soaked overnight. The next day, the samples were placed it in an electrothermal digestion apparatus for digestion. When 1 mL of solution remained left in the tube, it was removed and cooled. The volume was set to 25 mL and Na^+^, K^+^, and Ca^2+^ concentrations were determined using a flame atomic absorption spectrophotometer (AA-1800H; Shanghai Meixi Instrument Co., Ltd.); the standard solution configuration refers to the DB22/T 2344–2015 standard, and the linear correlation coefficient of the standard curve is 0.9998.

#### RNA Extraction, cDNA Synthesis, and Quantitative Reverse Transcription

The am 80 and nm 80 treatment groups showed the most significant differences in growth and physiology, so we selected these two treatment groups for qRT‒PCR analysis; fresh rice leaves were ground in liquid nitrogen, and RNA was extracted with an Omega Plant RNA Extraction Kit (Plant RNA Kit R6827). The integrity and purity were verified with a NanoDrop 2000c system (Thermo Scientific, Pittsburgh, PA, United States), and then reverse transcription was performed with a TAKARA-RR036A Reverse Transcription Kit (TAKARA RR036A PrimeScript™ RT Master Mix).

The primer sequences of related genes were cited from previous research results (Additional file 1: Table 1), and primers were synthesized by Sangon Biotech (Beijing). The reaction system was configured according to the template tracer-type dye quantification PCR assay kit (ChamQ SYBR Colour qPCR Master Mix (Low ROX Premixed), Vazyme). Each real-time fluorescence quantitative polymerase chain reaction mixture consisted of 15 μL of 2 × ChamQ SYBR qPCR Master Mix, 1.2 μL of primers, 2 μL of cDNA diluted 1:10, and 12.3 μL of ddH_2_O. The total reaction system was 30 μL. qRT‒PCR were conducted using a REFA40425 fluorescent quantitative PCR apparatus (Cottage Technologies Holdings Ltd.). The reaction conditions were selected according to the manufacturer's instructions (Additional file 1: Table 2) and the temperature required for the relevant primers. The relative expression level of transcript samples was analysed by the 2^−ΔΔct^ method; each treatment was repeated 3 times.

### Statistical Analysis

Microsoft Excel and SPSS 27 were used for data processing and statistical analysis. Plots were generated using Origin 2021b, PowerPoint, GraphPad Prism9, ggplot2, and ggbiplot2 packages in R software (4.2.1). The LSD test in one-way ANOVA was used to analyse the significance of the difference between the groups at the 0.05 level. In addition, a two-way ANOVA of salt, AMF compound inoculants, and their interaction was performed on the data.

## Supplementary Information

**Additional file 1.** Supplementary Tables and Figures.

## Data Availability

All data generated or analyzed during this study are included in this published article and its Additional files.

## Abbreviations

AMF: Arbuscular mycorrhizal fungi
Fm: *Funneliformis mosseae*
Pi: *Piriformospora indica*
Ar: *Agrobacterium rhizogenes*
ROS: Reactive oxygen species
Pro: Proline
SOD: Superoxide dismutase
POD: Peroxidase
qRT-PCR: Quantitative reverse transcription-Polymerase chain reaction
SPAD: Soil and plant analyzer develotrnent
*A*: Net photosynthetic rate
*E*: Transpiration rate
*C**i*: Intercellular CO_2_ concentration
*GH*_*2*_*O*: Stomatal conductance
MDA: Malondialdehyde
Gr: Glutathione reductase
PAL: Phenylalanine ammonia lyase
PCA: Principal Component Analysis
LFQ: Label-free quantification
ABA: Abscisic Acid
ASA-GSH: Ascorbate–glutathione
